# “Did I just do that?”—Six‐month‐olds learn the contingency between their vocalizations and a visual reward in 5 minutes

**DOI:** 10.1111/infa.12433

**Published:** 2021-09-27

**Authors:** Tamar Keren‐Portnoy, Helena Daffern, Rory A. DePaolis, Christopher M. M. Cox, Ken I. Brown, Florence A. R. Oxley, Mona Kanaan

**Affiliations:** ^1^ Department of Language and Linguistic Science University of York York UK; ^2^ AudioLab Department of Electronic Engineering University of York York UK; ^3^ Department of Communication Sciences and Disorders James Madison University Harrisonburg VA USA; ^4^ Interacting Minds Centre Aarhus University Aarhus Denmark; ^5^ Department of Music University of York York UK; ^6^ Department of Health Sciences University of York York UK

## Abstract

It has been shown that infants can increase or modify a motorically available behavior such as sucking, kicking, arm waving, etc., in response to a positive visual reinforcement (e.g., DeCasper & Fifer, 1980; Millar, 1990; Rochat & Striano, 1999; Rovee‐Collier, 1997; Watson & Ramey, 1972). We tested infants to determine if they would also change their vocal behavior in response to contingent feedback, which lacks the social, emotional, and auditory modeling typical of parent‐child interaction. Here, we show that in a single five‐minute session infants increase the rate of their vocalizations in order to control the appearance of colorful shapes on an iPad screen. This is the first experimental study to demonstrate that infants can rapidly learn to increase their vocalizations, when given positive reinforcement with no social element. This work sets the foundations for future studies into the causal relationship between the number of early vocalizations and the onset of words. In addition, there are potential clinical applications for reinforcing vocal practice in infant populations who are at risk for poor language skills.

## 1 INTRODUCTION

In a Dynamic Systems view of development, infants learn about the physical world by discovering contingencies between their own actions and resultant events. Over time, this process of discovery enables infants to learn to control their own actions and to repeat them at will with more predictable consequences. Thelen and Smith (1994) describe motor, as well as cognitive, development, as proceeding through exploratory, novel and variable behavior with no a‐priori solution that the system knows and is seeking to match. The move between old and new “attractors,” or behaviors that are stable is achieved by “stumbling” onto new behaviors through variable, unplanned action, upon encountering new situations or new contexts. Such exploration is what leads infants to new learning, both of new actions or behaviors and of their relationships to external events. Both in learning about one's own bodily functions and abilities and about the relationship between one's own body and external events, contingency, or contiguity in time brought about by a causal relationship are crucial (DeCasper & Carstens, 1981). Thelen and Smith (1994) stress the importance, in learning, of the time‐locked nature of multi‐sensory events: “The sensation of movement is as time‐locked with one external stimuli as the properties of the external stimuli are time‐locked with one another. Note that movement in this case includes not only large limb and trunk movements associated with locomotion and manipulation but also head and neck movements, and most significantly, eye movements (p. 193).” We would add to this list about the movements of the vocal apparatus (the parts of the body that produce speech sounds).

The current study described was aimed to test whether contingency learning could play a role in infants’ vocal development. When infants with normal hearing vocalize, they hear the auditory results of the actions they have just performed. This is contingency learning: learning about the contingencies between the motoric, proprioceptive, and auditory characteristics of different actions of the vocal apparatus. What we attempted to do in this study was to create a new contingency between an infant's vocalization and an appealing external event via a tablet application. If such a contingency could be learned by infants, this tablet application may be used to encourage more vocal exploration in infants, who are not very vocal.

Past research, starting with Piaget's observations of his children, when a chain tied to a rattle was placed in their hands (Piaget, 1954/1999), has shown infants to be capable of learning the contingencies between several kinds of actions and resultant external events, as measured by a modulation in their production of these actions: Newborns can learn to modulate the pause duration between sucking bursts on a dummy (pacifier)—both to increase and to decrease the pause duration—in order to hear their mother's voice (DeCasper & Fifer, 1980). Two‐month‐olds can learn to increase the amount of pressure they exert on a dummy to create auditory responses (Rochat & Striano, 1999), or to make small head movements in order to get a mobile to turn (Watson & Ramey, 1972). Six‐to‐8‐month‐olds can learn to increase the amplitude of arm movements (“arm excursion”), so that they cause a loose cord to become taut and activate a light and sound display (Millar, 1990). Rovee‐Collier and colleagues have shown in a series of studies that infants can also learn to increase the frequency of a motor behavior (leg kicking) due to positive conjugate reinforcement. The researchers tied the infant's foot with a ribbon to a mobile hanging above the infant's cot, such that when the infant kicked, the mobile would turn. Infants as young as 2 months of age learned to increase their kick rate in order to achieve the desired result of getting the mobile to turn and older infants learned to increase the frequency of hand banging in order to get a train to move on its tracks (Rovee‐Collier, 1997). In all these studies, the behavior upon that the contingency depends needs to be within the infant's repertoire. If the resultant event is positively reinforcing, the child learns to modulate the timing, intensity, amplitude, or rate of production of the behavior. In particular, an increase in the rate of production, by providing more practice opportunities, can lead to more automation and more control over (or repeatability of) the behavior, and a decrease in its random (or non‐context‐dependent) variability. Thus, the learning of the contingency may lead to the behavior becoming repeatable at will.

In all the studies described above, there were haptic, proprioceptive, and timing (simultaneity) cues for the existence of the contingency. The haptic cues involved the touch of the dummy in the mouth, the touch of the head on a pressure‐sensing pillow, the tug of the ribbon on the skin, the touch of the lever when banging on it, etc. But timing is crucial: The responses need to be time‐locked to the behaviors in order for the learning to occur (Skinner, 1948). In Millar’s (1990) study, which required 6–8‐month‐old infants to perform a big arm movement, response delays of 1 or 2 s led to reduced learning of the contingency, and a delay of 3 s disrupted learning completely.^1^ In the current study, we created a contingency whose initiation and frequency were infant‐dependent and that involved timing cues but no haptic cues, because the mechanism that provides the external event does not touch any part of the infant's body (unlike the dummy in the mouth, the ribbon on the foot, etc.). Like the Rovee‐Collier (1997) tasks, in which "the infants themselves are moving contingently with the mobile; the faster and harder they kick, the more vigorously the mobile will jiggle” (Thelen & Smith, 1994, p. 205), the task in the current study provided the child with analogous input to their own action in terms of timing, amplitude, and duration.

### 1.1 The current study

The contingency under investigation was implemented through a game, in which infants’ voiced vocalizations produced colorful shapes on the screen of a tablet device. The shapes appeared almost simultaneously with the vocalization and moved for as long as the vocalization continued, thus mirroring its duration, and their size was analogous to the amplitude of the vocalization, growing with rising amplitude and shrinking with a fall in amplitude. The primary research question was whether infants could learn that the display on the screen was related to their own vocalizations. If so, that would show us that infants can learn a self‐initiated contingency based uniquely on timing and “quantity” (amplitude and duration) cues, and crucially, one that is dependent on vocal behavior.

Although there exists literature on contingency learning in the context of infant vocalizations, such studies tend to focus on adult‐children interactions and how those affect infants’ vocalizations (e.g., Fagan & Doveikis, 2017; Goldstein & Schwade, 2008; Gros‐Louis et al., 2014; Messum & Howard, 2015). What is different about the contingency we are investigating is that it is not social. We are interested in studying and learning that takes place without the aid of adult “teachers.” In the studies mentioned above, infants are receiving what could be construed as social feedback as well as, if they are imitated by the adult, a “re‐interpretation” of their vocalizations through an adult language‐specific perceptual system and vocal tract. This type of learning, which can be seen as a kind of supervised learning, is different from the unsupervised learning that was the focus here.

Beyond the focus on the learning of a new contingency, this project opens up interesting avenues for further research: If creating a contingency‐learning scenario can encourage infants to vocalize more and, in particular, to babble more (i.e., to produce syllables containing consonant‐vowel sequences), and since increased babble may lead to an increase in automaticity and expertise, this in turn may facilitate the move into first words. Past research strongly suggests that babbling is a “tool,” which supports infants’ first word production (e.g., McCune & Vihman, 2001; McGillion et al., 2016; Oller, 2000; Oller et al., 1976; Vihman et al., 1985) and fundamentally changes the way the infants experience the world (DePaolis et al., 2011, 2013; Majorano et al., 2014). The contingency set‐up tested here may, therefore, have interesting clinical applications.

Our main research question in this project was: Will infants learn the contingency between their vocalizations and visual responses appearing on a screen? We expected such learning to be exemplified by an increase in infants’ vocalizations.

## 2 METHODS

Contingency learning was tested with 6.5‐month‐old infants. This age was chosen to coincide with the lower end of the typical period at which infants usually start to babble (age 6 months on average according to Oller, 2000; 7 or 8 months according to a newer systematic review by Morgan & Wren, 2018). Infants in the experimental group interacted with the game described above, which was implemented through an app we created, BabblePlay, described again below. Infants in the control group interacted with a similar but non‐contingent visual display.

### 2.1 Participants

The final samples contained 30 infants in each group (19 females in the experimental group and 12 in the control group). All infants were born full term, had normal hearing, and were typically developing, according to parental reports. Sixteen additional infants were recorded but taken out of the final sample (*N* = 7 in the experimental group, *N* = 9 in the control group). Reasons for exclusion in the experimental group were as follows: experimenter error (*N* = 2), mother interacting with infant during session (*N* = 2), an electronic toy playing music during session (*N* = 1), fussiness (*N* = 1), hiccups, which were picked up by the app (*N* = 1). Reasons for exclusion in the control group were as follows: experimenter error (*N* = 1), fussiness (*N* = 4). In addition, data from four additional control‐group infants waere not used because the experimental‐group infant they were yoked with (see below) was one of those excluded from the final sample. All infants were seen around age 6.5 months (mean age was 198 days in the experimental group [range 169–254 days] and 197 days in the control group [range 160–237]).

### 2.2 The BabblePlay App

**FIGURE 1:**
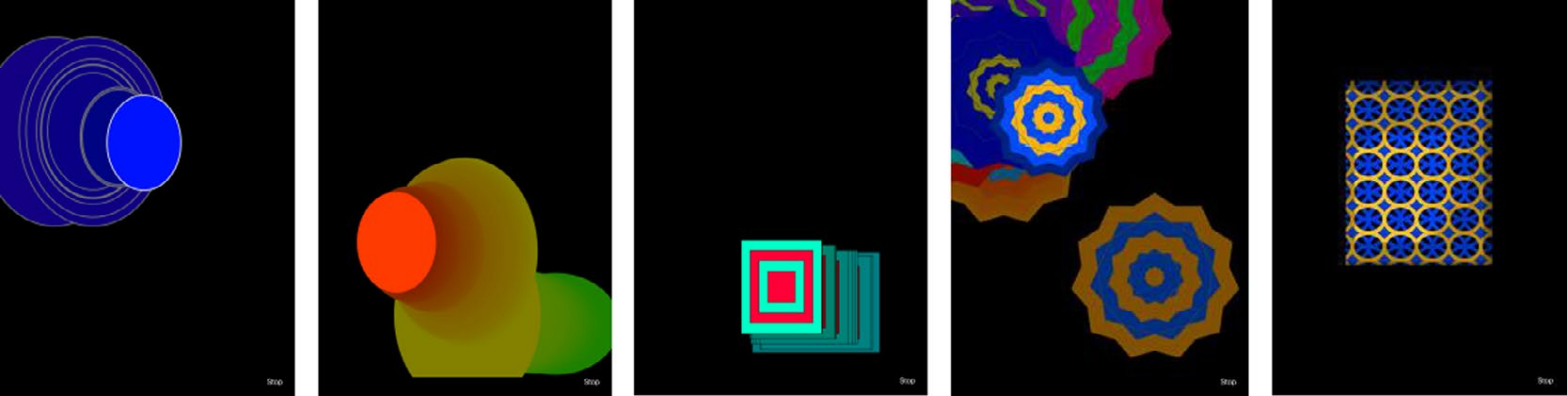
BabblePlay screenshots. *Note*. Examples of visual displays, showing the shape and its movement trajectory across the screen

BabblePlay responds to voiced infant vocalizations in real‐time with colorful moving shapes. After the app starts, the screen is initially blank (black), but within 160 ms of the start of an infant vocalization, a shape appears on the screen and continues to move for the duration of the vocalization. The shape, its colors, texture, initial size and location are random, but once it has appeared, its size changes with the loudness of a vocalization, growing larger with increased amplitude (see Figure 1 for some screen shots of the shapes the app produces). The app responds to all vocalizations within an infant's pitch range that are not too loud (so as not to encourage shouting and crying), not too high‐pitched (so as not to encourage shrieking), not too low‐pitched (so as to exclude potential interference from at least some adult speakers) and not too short or quiet. BabblePlay does not respond to environmental sounds such as bangs, rustling, and traffic noise (a demonstration video is available at URL https://www.york.ac.uk/babbleplay/). The correlations between a human judge and BabblePlay in identifying vocalizations ranged from .87 to .95 (for detailed information regarding the algorithms used to build BabblePlay and its reliability in identifying vocalizations as compared to human judges, see Daffern et al., 2020). BabblePlay turns off automatically after 5 min of use.

Interestingly, after we started developing the app we discovered that others have tried to develop similar tools in the past, that respond visually to infant vocalizations (see Angus & Kendall, 1988; Cohen, 1999a, 1999b; Fell et al., 2006, 2008). None, as far as we are aware, have been made use of, either for research or clinically, beyond a small pilot, probably due to their being hardware based, bulky, and difficult to use. Other attempts, such as Hailpern et al. (2009) used both visual and auditory combinations of reinforcement for vocalizations in older children (5–6 years old) with autism and/or Down syndrome, but the reinforcement in this case was not simultaneous with the vocalization (as far as we can tell), and so differs from our app in important ways.

### 2.3 Procedure

Sessions were initially conducted either in infants’ homes or in the laboratory, according to the family's preference. Later, for reasons of convenience, we switched to testing all the infants in the lab. Out of the 30 participants in each group, home tests were conducted with 10 infants in the experimental group and with 7 infants in the control group.

The infants sat in a rocker chair or on the carpet, with the parent next to them, propping them up from behind if necessary. Other people present, in addition to the parent/carer and infant, were an experimenter and, for the last 10 infants, an additional adult who operated a video camera. Each infant participated in a single session.

Each session began with a Solo‐Play trial, in which the infant played on their own for 5 min, with toys supplied either by the family or by the experimenter. During that time, the iPad was lying on the floor near the child, recording them, but unseen by them. If the toys rolled away from the infant, one of the adults handed them back to the child. The adults in the room were instructed not to talk or initiate interaction with the infant, but also not to ignore the infants by never returning gazes or smiles, so as not to unsettle them.

After the Solo‐Play trial, the infant was given a few minutes to relax, and then the 5‐min App trial began. During this trial the infants were all shown an iPad app. The iPad was held by the experimenter who moved the screen so that it would face the infant as much as possible, since the infants often looked away or turned away from the iPad. The experimental‐group infants were shown BabblePlay and their vocalizations were recorded. We then replayed each infant's recorded sound file to the app in the lab and created a “video” of BabblePlay's display that resulted from the visual responses to that infant's vocalizations. Each control‐group infant was “yoked” to one experimental‐group infant and shown that infant's video. In the control‐group trials, another iPad with BabblePlay installed on it was placed on the floor where it was not visible to the control‐group infant and recorded their vocalizations.^2^

In two cases (one in each group) the Solo‐Play trial did not last the entire 5 min because the infants lost interest in playing. For those sessions, the number of vocalizations is estimated by extrapolation, assuming that the rate of vocalizing per minute would have remained the same. In other cases, BabblePlay continued to respond to infant's vocalizations for a few additional seconds after the 5 min had elapsed. This happened more often in the experimental than in the control group. For sessions longer than 5 min, vocalizations were cropped at 5 min. All values used in the tests reported below are those corrected to 5 min.

BabblePlay sometimes recorded and responded to noises that were not infant vocalizations, such as rare adult vocalizations (e.g., “oops,” “sorry”) or a toy noise. In other cases, it responded to noises produced by the infant, which were not vocalizations, for example, burps, coughs, and sneezes. We treated those as noise in the data and made no attempt to correct for them (see Appendix A for cases in which we diverged from the described protocol).

In all the trials, a video‐recorder filmed the infants’ faces (this footage is not used in this paper). In the last 10 sessions in each group, an additional wide‐angle video camera was used to capture the experimenter's face, the infant's face and the iPad screen, so that we could address several potential concerns around possible confounding variables (see tests for internal validity A–D, below). Given that the experimenter who held the iPad during the App trials could not be blind to the infant's group membership, we wanted to test whether that affected their behavior towards the infants in the two groups, resulting in a possible experimenter effect. We, therefore, tested whether there is evidence that the facial cues they imparted to the infants in the two groups may have been systematically different (internal validity tests A and B). The infants interacting with the app were surrounded by several potential interactants and may have responded to the two conditions systematically differently, which could have resulted in a subject effect. We, therefore, also tested the infants’ degree of engagement with these adults’ faces during the session (internal validity tests C and D).

The present study was conducted according to guidelines laid down in the Declaration of Helsinki, with written informed consent obtained from a parent or guardian for each child before any assessment or data collection. All procedures involving human subjects in this study were approved by the Language & Linguistic Science ethics committee at the University of York.

#### 2.3.1 Operational research questions

1. Do infants in the experimental group vocalize more than infants in the control group during the App trial (after controlling for baseline volubility, based on the Solo‐Play trial)?
2. Do the experimental‐group infants show evidence of learning within the App trial? That is, do infants in the experimental group, but not those in the control group, show an increase in the number of vocalizations between the first and second half of the trial? We postulated this question in a post hoc manner following exploration of the vocalization counts of the last 10 infants in the experimental group. We have taken this exploration into account in the ensuing analysis.

Research Questions 1 and 2 were tested using the frequency of infant vocalizations over the 5‐minute Solo‐Play and App trials, as counted by the app, as the dependent variable.

#### 2.3.2 Testing the internal validity of the findings

To account for possible confounding variables, we assessed whether the source of reinforcement for the experimental group could be the faces of the people involved in the running of the trial rather than the shapes on the screen (i.e., an experimenter effect). We, therefore, tested the following:

- whether the experimenter's facial expression was judged to be more encouraging when conducting sessions with the experimental than with the control group.
- whether infants in the experimental group increased their looks to the experimenter's (or filmer's) face between the first and second half of the trial.

Conversely, it is possible that the vocalizing behavior of the control group could be more related to attempts to interact with and seek attention from the adults in the room than to the shapes on the screen. To examine if this could be a possible confounding variable for explaining the frequency of vocalizations in the control group (i.e., a subject effect) we tested the following:

- whether infants in the control group increased their looks to the experimenter's (or filmer's) face between the first and second half of the trial.
- whether control‐group infants looked more at faces overall (both the experimenter's and the filmer's) than did experimental‐group infants.

The tests for internal validity were tested on the final 10 infants seen in each group, based on wide‐angle camera footage of the App trial. The dependent variable for test A was an assessment by a naive coder, based on the experimenter's facial expression, of whether the infant being tested was in the control or experimental group. If coders who were blind to the true group assignment could not guess the correct group, we deem that an indication that the experimenter was not systematically giving different cues to infants according to their group assignments. The dependent variables for tests B–D were the number of looks and duration of look to the experimenter's face, the camera (or the filmer's face), or the iPad, during the App trial.

#### 2.3.3 Obtaining the dependent measures from the video footage

1. Assessment of experimenter expression. We cropped the experimenter's face out of the footage of the entire scene. Because in the control group (but not in the experimental group), the experimenter occasionally looked down at the BabblePlay app on the floor to see whether it was still recording, we cut out downward looks from the control‐group videos, and an identical number of random sections from the experimental‐group video that this particular control infant was yoked to, so that both would contain the same number of discontinuities. The clips were randomly named, and the coders were blind to the group assignment of the infants. The coders’ task was to assess whether the infant in that trial was a control infant or an experimental infant, by the degree to which the experimenter's face seemed encouraging (for instance by gauging how often the experimenter smiled). The coders were told that there were identical numbers of clips from each of the groups (*n* = 8 from each group, *n* = 16 in total for the first coder. The other coder was erroneously given only *n* = 12 clips, and those were not balanced in number between the two groups: Five were from experimental‐group trials and seven from control‐group trials. The coder did not know that the number of files from the two groups were not identical, as the error was only discovered after they had finished making their judgements).
2. Number of looks and duration of look to the experimenter's face, the camera (or the filmer's face), or the iPad. This was measured for the final 10 infants seen in each group—based on wide‐angle camera footage of the App trial, using ELAN (Version 5.0.0) [Computer software], Nijmegen: Max Planck Institute for Psycholinguistics. Retrieved from: https://tla.mpi.nl/tools/tla‐tools/elan/ (see Sloetjes & Wittenburg, 2008), on each half trial separately. Coders did not know that half (first or second) was being coded at any given time. Coders reported that the main cue they used in judging look direction (i.e., to the experimenter, the filmer, or the iPad) was the direction in which the baby's eyes were pointing. They used the baby's head, hand or body movement as additional cues. For infants in the experimental group, all clips were first coded by a main coder whose judgments were used in the statistical analyses reported below, and then re‐coded by one of two additional coders for reliability. The mean proportion of agreement between coders regarding direction of look for infants in the experimental group was robust—0.80 per clip (range 0.64–0.94). For looks on which there was an agreement, the mean difference in the start time identified was 369 ms (range 153–865). As we assessed the above proportion of coder agreement for this task to be sufficiently high, the coding of the direction and duration of infants’ look in the control group was conducted by a single coder (the fourth author).

## 3 RESULTS

All analyses were carried out using SPSS 25 (2017), apart from Research Question 1, which was analyzed using STATA 14 (2015), and the internal validity tests B–D, which were analyzed using R 4.0 (R Core Team, 2013) in R Studio 1.4 (RStudio Team, 2021). All *t*‐tests were run as two‐tailed tests, assuming a significance value of 0.05.

Table 1 (left 2 columns) shows descriptive statistics for the two groups in the full sample (“full set” in the table), including the infants’ sex and age. As can be seen, the average ages of the infants in the two groups were almost identical.

**TABLE 1:** Group characteristics and number of vocalizations as tallied by the app

Group	Full set		Smaller set	
	Experimental	Control	Experimental	Control
*N*	30	30	20	20
*N* females	19	12	12	9
Age in days				
*M*	197.53	196.6	198.2	195.2
*SD*	19.72	21.17	20.50	23.20
Number of vocalizations in solo‐play trial				
*M*	22.43	20.33	20.85	19.85
*SD*	18.26	17.47	15.77	17.12
Number of vocalizations in app trial				
*M*	29.23	24.33	26.00	26.40
*SD*	20.88	16.42	17.69	14.28

Research Question 1: Do infants in the experimental group vocalize more than infants in the control group during the App trial (after controlling for baseline volubility, based on the Solo‐Play trial)?

In order to address Research Question 1, we conducted a linear regression analysis of the frequency of infant vocalizations in the App trials (the dependent variable), adjusting for the pairing (or yoking) mentioned above (similarly to pairing used in twin studies, see e.g., Carlin et al., 2005). To measure the effectiveness of BabblePlay versus the non‐interactive app, we controlled for group membership as an indicator (dummy) variable. In order to account for possible differences by child in baseline volubility (as per Vickers & Altman, 2001), we controlled for the Solo‐Play trial vocalization frequency as one of the predictors in the linear regression model; we also explored whether there is an interaction between these vocalizations and group membership.

Based on the regression analysis results (see Table 2 for details), we found no evidence of a statistically significant difference in the mean number of vocalizations between the experimental and control groups (coefficient = 0.624, *p* = .90), when controlling for the number of Solo‐Play vocalizations. Furthermore, we found no evidence of an interaction between the Group and the number of Solo‐Play vocalizations (coefficient = 0.106, *p* = .61).

**TABLE 2:** Linear regression analysis results for number of vocalizations

	Coefficient^a^	Robust Standard Error	*p*‐value	95% Confidence Interval	
Group	0.624	4.859	.899	−9.314	10.563
Number of Solo‐Play vocalizations	0.464	0.120	.001	0.219	0.709
Interaction term (Number of Solo‐Play vocalizations and Group)	0.106	0.207	.614	−0.318	0.529
Constant term	18.200	2.857	.000	12.358	24.042
*R* ^2^ = 19.55%					

Note

Adjusted coefficients, standard errors, *p*‐values and 95% confidence intervals are presented.

^a^ Adjusted coefficients controlling for group, solo‐play vocalizations, and allowing for an interaction between them. The model also takes into account the yoking of the infants.

Research Question 2: Do the experimental‐group infants show evidence of learning within the App trial? Do infants in the experimental group, but not the control group, increase the number of vocalizations between the first and second half of the trial?

We again used the frequency of infant vocalizations to address Research Question 2. Because infants differed greatly in how much they vocalized, for both groups, we converted the vocalization counts in the App trials to proportion scores. These were computed as the proportion of vocalizations produced in the second half of the App trial to the total number of vocalizations produced in the App trial. Proportions over 0.5 indicate an increase in vocalizations in the second half. Proportions of 0.5 or below indicate no such increase.

We used paired *t* tests to compare the proportion scores between the two groups. To further investigate the source of the difference between the groups, we followed this test with two one‐sample *t* tests, to see whether the proportion of vocalizations in the second half of the session was different from 0.5 in each group. This analysis was exploratory and the statistical significance tests can only be taken as suggestive, because we conducted it after exploring the vocalization patterns of the last 10 infants in the experimental group.

In order to differentiate between our initial exploratory analysis and the post hoc analysis, we will refer to our original sample of 30 pairs of infants as the “full set;” one pair of which had to be removed from some paired analyses due to the fact that the control‐group infant in that pair had no vocalizations recorded in the App trial. As we had explored the vocalization counts for the last 10 infants in the experimental group prior to running the analysis addressing Research Question 2, we created a sub sample, henceforth referred to as the “smaller set,” of only those infants whose data that we have not explored. This smaller set contained the 30 control‐group infants and the remaining 20 experimental‐group infants. However, in this smaller set only 40 infants were yoked, which resulted in 20 pairs. We re‐ran the tests used to address Research Question 2 on the remaining 20 pairs of infants in the smaller set.

We start by describing the tests on the full set. As can be seen from Table 3 (2 left columns), infants in the control group were equally likely to increase the frequency of vocalization between the first and second halves of the App trial as they were to decrease it (47% vs. 53%, respectively). Infants in the experimental group, however, were much more likely to increase the frequency of vocalizations from the first to the second half than to decrease it (83% vs. 17%, respectively).

**TABLE 3:** Change in frequency of vocalizations from 1st to 2nd half

Group	Full set		Smaller set	
	Experimental	Control	Experimental	Control
Decrease or no change – Number of infants	5	15	4	10
Increase – Number of infants	25	14	16	9
Proportion increase	.83	.47	.80	.47

**FIGURE 2:**
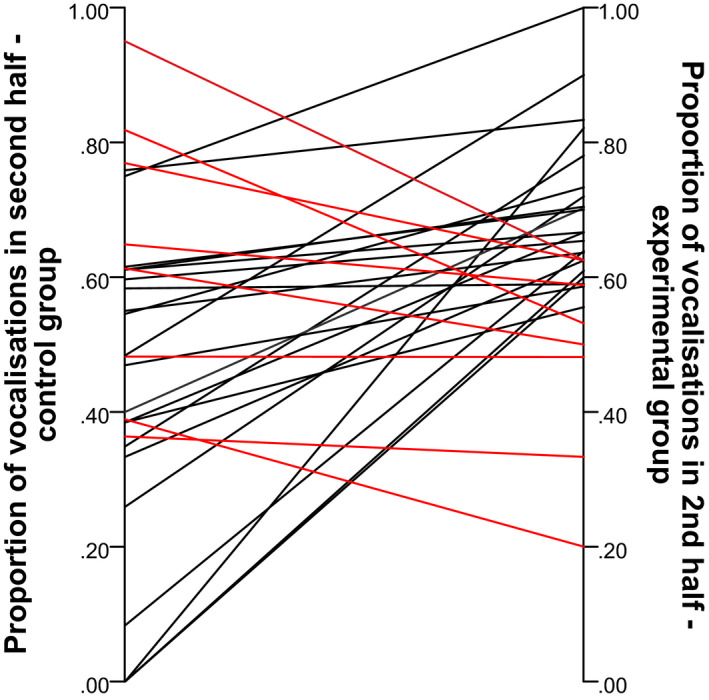
Differences in the proportion of vocalizations in the second half of the app trial by infant pairs (full set). *Note*. Black lines, which are higher on the right side than on the left, depict the pairs in which there was a higher proportion of vocalizations in the second half of the trial for the experimental‐group infant than for its yoked control‐group infant. Red lines, which are higher on the left than on the right, show the *n* = 8 pairs (out of 19), which exhibit the opposite pattern

**FIGURE 3:**
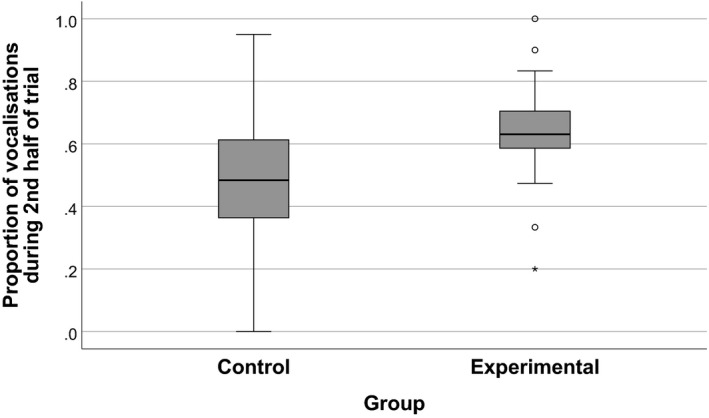
Distribution of proportions of vocalizations in the 2nd half of the session (full set). *Note*. Mean proportion by chance would be 0.5. Circles indicate outliers (more than 1.5 times the interquartile range as measured from the closest quartile) and asterisks indicate extreme outliers (more than 3 times the interquartile range as measured from the closest quartile)

In 21 of 29 pairs the proportion of vocalizations in the second half was higher for the experimental‐group infant than for the control‐group infant, indicating that in the experimental group, there was a greater increase in vocalizations in the second half of the App trial (see Figure 2). The distributions of the proportions and of the proportion differences were approximately normal (as assessed by a Shapiro–Wilk normality test and via inspection of a Q–Q plot). The difference in proportions was statistically significant based on a paired *t*‐test: *M*(difference) = .16, *t*(28) = 3.195, *p* = .003. Furthermore, in the control group, the mean proportion (*M* = 0.48) was not significantly different from 0.5 (*t*(28) = .0.528, *p* = .6). However, in the experimental group, the mean proportion (*M* = 0.64) was statistically significantly different from 0.5 (*t*(29) = 4.779, *p* < .001); see Figure 3 for the distribution of proportions in each group relative to the 0.5 value.

We re‐ran the tests again on the smaller set. The results mirror those for the full set. In this set too, the assumption of normality was met (as assessed by a Shapiro–Wilk normality test and via inspection of a Q‐Q plot). In 14 of 19 pairs the proportion of vocalizations in the second half was higher for the experimental‐group infant than for the control‐group infant, and all the *t* tests were statistically significant, as in the full set (see Appendix B for the details of the tests and the related figures).

### 3.1 Internal validity tests—testing for confounding variables

Test A: Is the experimenter's facial expression judged as more encouraging when conducting sessions with the experimental than with the control group?

We calculated the success rate of the coders at guessing the infants’ group based on the visual cues given by the experimenter and compared their success rate to what is expected by chance, using a binomial distribution.

The first coder assigned the correct group in seven out of 16 clips. The probability of 7 successes out of 16 trials, with a .5 probability of success (using a binomial distribution), is *p* = .40. The second coder assigned the correct group in six out of 12 clips. The probability of 6 successes out of 12 trials, with a .58^3^ probability of success (using a binomial distribution), is *p* = .38. As the success rates are not significantly different from chance, there is no indication that the experimenter gave any visual cues to the infants with their facial expression that would encourage more vocalizations in one group than in the other.

Test B: Are infants in the experimental group increasing their looks to the experimenter's (or filmer's) face between the first and second halves of the trial?

Paired *t* tests were conducted on the difference between the first and second half of the trial for each infant. These parametric tests were applied on log‐transformed distributions so as to satisfy the assumption of normality. The statistics and the *p*‐values are presented in Table 4. Mean differences of the frequency of looks: to experimenter: *M*(difference) = 2.1, *p* = .28; to camera/filmer: *M*(difference) = −1.1, *p* = .62; to iPad: *M*(difference = 2.8, *p* = .07. Mean differences of duration of looks: to experimenter: *M*(difference) = 7.33, *p* < .01; to camera/filmer: *M*(difference) = 0.15, *p* = .46; to iPad: *M*(difference = −6.76, *p* = .99). These indicate that there is lack of evidence of a statistically significant difference in the number and duration of looks between the two halves of the App trial for the 10 infants of the experimental group for whom these measurements were recorded, apart from infants’ durations of looks toward the experimenter, which show a significantly lower duration during the second half of the trial. This suggests that it is unlikely that the increase in vocalizations between the two halves in this group was a result of reinforcement by the adults (experimenter and filmer) involved in the trial. There is also lack of evidence of a statistically significant difference in the mean number and duration of looks between the two halves of the App trial for looks towards the iPad screen. We return to this point in the Discussion.

**TABLE 4:** Number and duration of looks in the two app trial halves ‐ experimental group (*n* = 10)

	First half		Second half		*T* statistic	*p*‐value
	*M*	*SE*	*M*	*SE*		
Looks to experimenter						
Frequency	5.20	1.70	3.10	0.74	1.156	.278
Duration	11.50	4.50	4.17	1.35	3.553	.006
Looks to Camera/filmer						
Frequency	5.60	1.43	6.70	1.75	−.510	.623
Duration	11.59	4.30	11.44	4.00	.772	.460
Looks to iPad						
Frequency	16.70	1.27	13.90	1.54	2.067	.069
Duration	55.50	8.82	62.26	11.02	−.016	.987

Note

The paired *t* tests have been applied on log‐transformed distributions so as to satisfy the assumption of normality (as assessed by a Shapiro–Wilk normality test and via inspection of a Q–Q plot).

Test C: Are infants in the control group increasing their looks to the experimenter's (or filmer's) face between the first and second half of the trial?

We ran paired *t*‐tests on the difference in number and duration of looks between the first and second half of the trial for each infant in the control group. The statistics and the *p*‐values are presented in Table 5. Mean differences of the frequency of looks: to experimenter: *M*(difference) = 2.10, *p* = .95; to camera/filmer: *M*(difference) = 1.9, *p* = .95; to iPad: *M*(difference = 9.90, *p* = .57. Mean differences of duration of looks: to experimenter: *M*(difference) = 1.54, *p* = .62; to camera/filmer: *M*(difference) = 1.93, *p* = .42; to iPad: *M*(difference = 5.90, *p* = .47). As can be seen, no significant differences were found.

**TABLE 5:** Number and duration of looks in the two app trial halves—control group (*n* = 10)

	First half		Second half		*T* statistic	*p*‐value
	*M*	*SE*	*M*	*SE*		
Looks to experimenter						
Frequency	2.10	0.57	1.9	0.41	−.069	.947
Duration	1.54	0.32	1.71	0.31	−0.514	.620
Looks to Camera/filmer						
Frequency	1.90	0.48	2.30	0.86	0.072	.945
Duration	1.93	0.64	1.22	0.29	0.843	.421
Looks to iPad						
Frequency	9.90	0.86	9.70	1.18	0.594	.567
Duration	5.90	0.87	6.41	0.78	−0.760	.466

Note

The paired *t*‐tests have been applied on log‐transformed distributions so as to satisfy the assumption of normality (as assessed by a Shapiro–Wilk normality test and via inspection of a Q‐Q plot).

Test D: Do infants in the control group look more at faces overall (both the experimenter's and the filmer's) than infants in the experimental group?

The two‐tailed paired *t* test comparing infants’ frequency of looks in the 5‐min’ session toward faces in the control group (*M* = 8.20, *SD* = 4.57) and in the experimental group (*M* = 20.8, *SD* = 11.97) showed that the yoked infants in the control group exhibited significantly fewer looks (*t*(9) = 2.813, *p* = .020). Relatedly, a two‐tailed paired *t* test showed the duration of these looks in the control group (*M* = 6.40, *SD* = 3.10) to be significantly shorter than in the experimental group (*M* = 38.97, *SD* = 32.19) (*t*(9) = 3.297, *p* = .009).

## 4 DISCUSSION

Our study investigated whether infants could learn the contingency between their own vocal behavior and an external time‐locked visual response. We will start by summarizing our findings in relation to the research questions that were raised in the Introduction. We will then discuss the implications of our findings for infant learning and for potential clinical applications.

We asked whether the infants can learn the contingency between their vocalizations and the app's visual responses. The first test, in which we compared the experimental to the control group in terms of the frequency of vocalizations in the App trial, while controlling for frequency of vocalizations in the baseline Solo‐Play trial, showed no evidence of a difference between the groups. However, when we compared the change in vocalizing behavior between the two groups over the course of the App trial, we saw that the experimental‐group infants increased the frequency of their vocalizations from the first half of the trial to the second half, while the control‐group infants did not. That indicates that the experimental group learned the contingency between their vocalizations and the resultant shapes on the screen. The control group showed no such systematic change. We explored an alternative explanation for the change in the vocalizing behavior of the experimental‐group infants, namely that it took place in response to social cues or reinforcement provided by people, rather than by the app. However, neither test supported nor the interpretation that the infants were responding to reinforcement in the form of social cues. We, thus, think that these alternative explanations of the findings are not supported by the data, although we cannot rule out the possibility that other social cues not measured here could have been differentially present in the two groups. One limitation of the current study was that we could not analyses the change in looks to the iPad as an indication of learning the contingency. The reason for that is that neither an increase in duration nor in the number of looks to the iPad would necessarily show learning. To judge learning based on look behavior, future studies with the app could assess whether there is an increase in anticipatory looks toward the iPad prior to or in tandem with vocalizing, in anticipation of the shapes appearing.

It is interesting that there was no evidence for a difference in the frequency of vocalizations between the two groups, as per the first test (Research Question 1). We predicted that infants who received reinforcement from the app would show a larger increase in the amount of vocalizations from the Solo‐Play trial to the App trial than those whose vocalizations received no such reinforcement. Such a difference was not found. We also tested whether it is possible that the infants in the control group became bored more quickly because they were watching a non‐interactive video, such that some of their vocalizations may have been attempts to elicit attention or responses from the adults in the room in this unfamiliar situation, in which several adults are looking at them but are not interacting with them. However, the control‐group infants did not increase their looks to the adults’ faces as the session progressed. They also looked less at the experimenter's and filmer's faces than did the experimental‐group infants. It is possible that they were attempting to look at their parent/carer's face, but we could not code for this. It is also possible that they were vocalizing to entertain themselves and not in order to engage others in interaction.

The experiment described here extend the previous findings regarding contingency learning by infants by investigating an intermodal, real‐time contingency between infants’ vocalizations and a visual response, where the timing and, crucially, the initiation of the contingent responses both depend entirely on the self‐initiated actions of the infants (i.e., the screen remains black if the infants do not vocalize). The learning of this contingency requires infants to anticipate the visual effects of their self‐initiated vocalizations and places infants in full control of exploring the contingency under investigation.

In addition, this is the first study we are aware of to demonstrate real‐time vocal learning outside of a social‐interactional situation. Social feedback is known to reinforce prelinguistic vocalizations, and it has been suggested that an infant's vocal behavior becomes more mature in response to adult speech (Goldstein & Schwade, 2008; but see Fagan & Doveikis, 2017, who do not find evidence for such an effect). Others have also shown the importance for later language learning of parental contingent responses to children (e.g., McGillion et al., 2013). Clearly, children learn a lot from their social partners. In this study, we have shown infants changing their vocal behavior based on their own production practice and experience: the infants produce sounds and perceive their effects without the mediation, interpretation or mirroring of an interlocutor's responses. The two mechanisms, learning based on own past behavior, and learning based on others’ responses to own past behavior, are likely to be complementary rather than competing.

The ability of infants to learn a contingency when engaged in screen time has provocative implications for the current debate over the impact of visual displays on infant development. In the lively debate about the use of touchscreens with young children and infants, decisions and advice should be based upon studies, such as this one. The concerns around mobile device use with young children, while meriting further research, are not currently based on solid empirical evidence (see Bedford et al., 2016).

Now that we have seen that such learning is possible, we see two potential directions for further research: Firstly, there have now been several studies, which found that mastering the production of consonants, as measured by frequent and sustained production of such consonants, is correlated with earlier word production (McCune & Vihman, 2001; McGillion et al., 2016). If infants can engage with BabblePlay for extended periods of time, and that would lead them to vocalize more than they would have otherwise, and if they have already begun to produce consonants, then the use of BabblePlay is likely to increase their consonant production, thus bringing forward their age for consonant mastery. We will, thus, be able to experimentally test the effects of increased vocalizing on later productive lexicons, with such an increase being achievable through self‐exploration, without involving the confounding (although undoubtedly highly beneficial) variable of increased parental interaction. Secondly, this work sets the foundations for exploring the benefits of technology for reinforcing vocal practice in infant populations who are at risk for poor language skills (e.g., low socioeconomic status infants: Hart & Risley, 1995), or in young children with language delay who find social interaction challenging (e.g., autistic children. See Clifford & Dissanayake, 2008, regarding social interaction and McDaniel et al., 2018, regarding language delay). This type of intervention is particularly timely for autistic children: McDaniel et al.’s meta‐analysis (2018) finds a strong relationship between vocalizing and expressive lexicon in autistic children and recommends further research into this relationship to guide future interventions. We are now embarking on a longitudinal study to test whether indeed this contingency‐learning set‐up can lead to earlier word production in typically developing infants from low socioeconomic status homes and are also hoping to test its appropriateness for autistic children.

## CONFLICT OF INTEREST

The authors declare no conflicts of interest with regard to the funding source for this study.

## Figures and Tables

**FIGURE A1:**
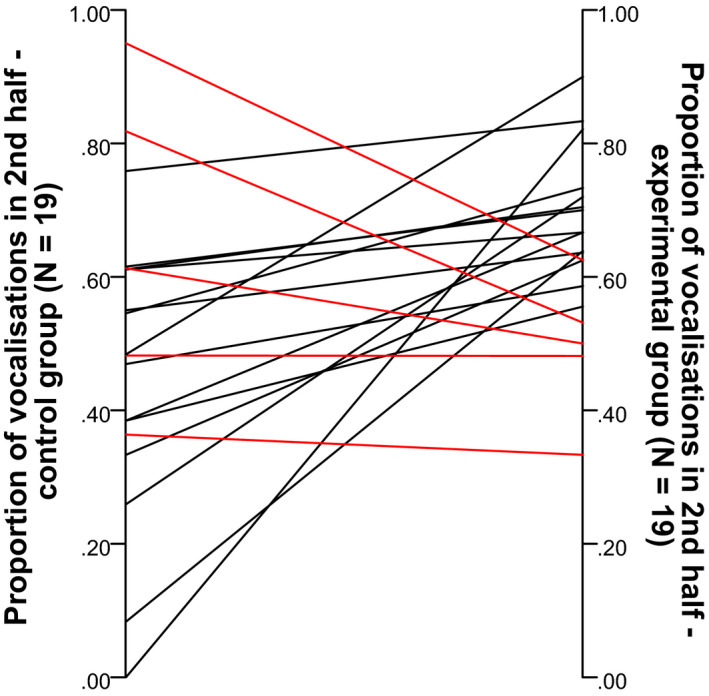
Differences in the Proportion of Vocalizations in the Second Half of the App Trial by Infant Pairs (Smaller Set). *Note*. Black lines, which are higher on the right side than on the left, depict the pairs in which there was a higher proportion of vocalizations in the second half of the trial for the experimental‐group infant than for its yoked control‐group infant. Red lines, which are higher on the left than on the right, show the *n* = 5 pairs (out of 19), which exhibit the opposite pattern

**FIGURE A2:**
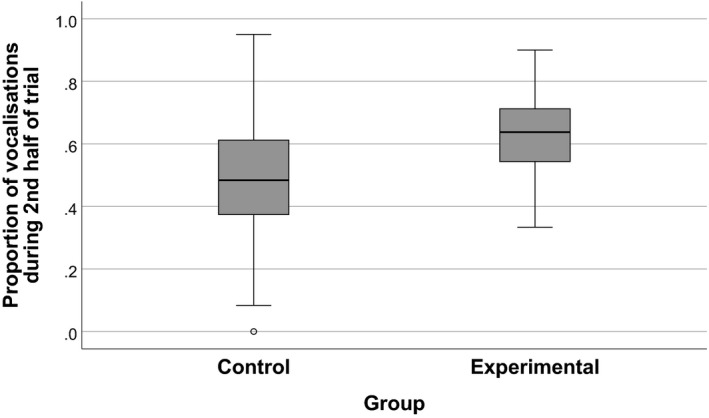
Distribution of Proportions of Vocalizations in the 2nd Half of the Session (Smaller Set). *Note*. Mean proportion by chance would be 0.5. The circle indicates an outlier (more than 1.5 times the Interquartile range as measured from the lower quartile)

## Footnotes

^1^
Wang et al. (2012) tested whether 6‐ and 8‐month‐olds use gaze to trigger the appearance of a new visual stimulus. Once infants focused their gaze on a dot on a screen, a new stimulus appeared. Anticipatory looking times to the onset of the new visual stimulus decreased over time, suggesting the contingency was learned. However, unlike the other tasks described so far, in which the initiation of the event that leads to the contingency and its frequency are wholly dependent on the infant, here the dot serves as a stimulus to instigate infant response.

^2^
Unfortunately when the iPad with BabblePlay was on the floor (in the App trials for the control group and in the Solo‐Play trials) its microphone was further away from the infants’ mouths than when it was held up in front of them, as in the App trials for the experimental group. To assess the effect of distance on the app's ability to identify vocalisations, we played ten infants’ sound files to the app from 40 cm and again from 100 cm. On average, the app identified 3.3 more vocalisations when closer: The mean number of vocalisations identified in the two identical sets of sound files was 34.9 (*SD* = 27.6) at 40 cm, and 31.6 (*SD* = 26.9) at 100 cm. This was controlled for in the analysis of Research Question 1, the only analysis that compared the number of vocalisations between two trial types.

^3^
Due to the imbalanced set of clips given to this coder.

## APPENDIX A

### Divergences from the planned protocol

1. In some cases, the infants became fussy and began crying before we could run the App trial. In such cases, we conducted a second session. We used data from a second session for 4 infants out of the 30 in the experimental group and for 1 infant out of the 30 in the control group. However, in order to give all the infants the same one‐off experience with the app, we avoided conducting a second session if a child had already started an App trial in the first session. In all cases but one, the infant only encountered the app in the second session. Only in a single case (in the experimental group) did we use the second session after the infant had begun an App trial in the first session but completed only 3 s of it.
2. In two cases, one in each group, an infant had two Solo‐Play trials on a single day, one, which had to be stopped, and another, which went fine. We used the second one and the App trial that followed it.
3. One infant in the control group was left in the sample even though they were shown the “App video” twice in the session. In the first attempt, which took around 30 s, the infant was very inattentive. The second trial lasted for the entire 5 min.

## APPENDIX B

### Results of tests run on the smaller set (*N* = 20 pairs)

Descriptive statistics for the two groups in the smaller set, including the infants’ sex and age, can be seen in Table 1 (right 2 columns). As can be seen in Table 1, the mean ages in the two groups are nearly identical in the smaller set, as was the case for the full set.

A paired *t* test comparing proportion scores between the two groups showed a significant positive difference between the proportions within the pairs, signifying a higher proportion in the experimental group, as expected: *M*(experimental) = .65, *M*(control) = .49, *M*(diff) = .16, *t*(18) = 2.429, *p* = .026, two‐tailed (see Figure A1). We followed this test with two single‐sample *t* tests, to see whether the proportion of vocalizations in the second half of the session was different from 0.5 in each group. In the control group, the mean proportion (*M* = 0.49) was not significantly different from 0.5 (*t*(18) = 0.180, *p* = .859, two‐tailed). In the experimental group, the mean proportion (*M* = 0.65) was significantly different from, and as expected, higher than, 0.5 (*t*(19) = 4.498, *p* < .001, two‐tailed) (see Figure A2 for the distribution of proportions in each group relative to the 0.5 value).
